# Insights into Drivers of Liking for Avocado Pulp (*Persea americana*): Integration of Descriptive Variables and Predictive Modeling

**DOI:** 10.3390/foods10010099

**Published:** 2021-01-06

**Authors:** Luis Martín Marín-Obispo, Raúl Villarreal-Lara, Dariana Graciela Rodríguez-Sánchez, Armando Del Follo-Martínez, María de la Cruz Espíndola Barquera, Jesús Salvador Jaramillo-De la Garza, Rocío I. Díaz de la Garza, Carmen Hernández-Brenes

**Affiliations:** 1 Tecnologico de Monterrey, Escuela de Ingenieria y Ciencias, Ave. Eugenio Garza Sada 2501, Monterrey, Nuevo Leon 64849, Mexico; A00815241@itesm.mx (L.M.M.-O.); raulvl@tec.mx (R.V.-L.); dariana@tec.mx (D.G.R.-S.); A00822822@itesm.mx (J.S.J.-D.l.G.); rociodiaz@tec.mx (R.I.D.d.l.G.); 2 SensoLab Solutions, Centro de Innovacion y Transferencia Tecnologica (CIT2), Ave. Eugenio Garza Sada 427, Monterrey, Nuevo Leon 64849, Mexico; 3 ALFA, Centro de Tecnologia de Sigma Alimentos, Apodaca, Nuevo Leon 66629, Mexico; adelfollo@sigma-alimentos.com; 4 Fundacion Salvador Sanchez Colin, CICTAMEX, S. C. Ignacio Zaragoza 6, Coatepec Harinas, Estado de Mexico 51700, Mexico; mespindolab@gmail.com

**Keywords:** avocado, cultivars, preference mapping, sensory evaluation, sensory descriptive analysis, consumer science

## Abstract

Trends in new food products focus on low-carbohydrate ingredients rich in healthy fats, proteins, and micronutrients; thus, avocado has gained worldwide attention. This study aimed to use predictive modeling to identify the potential sensory drivers of liking for avocado pulp by evaluating acceptability scores and sensory descriptive profiles of two commercial and five non-commercial cultivars. Macronutrient composition, instrumental texture, and color were also characterized. Trained panelists performed a descriptive profile of nineteen sensory attributes. Affective data from frequent avocado adult consumers (*n* = 116) were collected for predictive modeling of an external preference map (*R*^2^ = 0.98), which provided insight into sensory descriptors that drove preference for particular avocado pulps. The descriptive map explained 67.6% of the variance in sensory profiles. Most accepted pulps were from Hass and Colin V-33; the latter had sweet and green flavor notes. Descriptive flavor attributes related to liking were global impact, oily, and creamy. Sensory drivers of texture liking included creamy/oily, lipid residue, firmness, and cohesiveness. Instrumental stickiness was disliked and inversely correlated to dry-matter and lipids (*r* = −0.87 and −0.79, respectively). Color differences (∆E_ab_*) also contributed to dislike. Sensory-guided selection of avocado fruits and ingredients can develop products with high acceptability in breeding and industrialization strategies.

## 1. Introduction

Avocado fruits are now of high economic value, and thus, the food industry is showing a remarkable interest to enhance the production and processing of this crop [1,2]. According to the Statistical Division of the Food and Agriculture Organization of the United Nations (FAOSTAT), Mexico is the major producer and exporter of avocado worldwide. In 2018, Mexico’s avocado production was 2,184,663 t, with a harvest area of 206,389 ha, representing a 2.17-billion-dollar market [3]. Nutritional characterization of avocado fruit identified many functional compounds, which include unsaturated fatty acids, vitamin E, tocopherols, ascorbic acid, B vitamins, carotenoids, potassium, phenols, antioxidants, phytosterols, acetogenins, and its derivatives containing a furan ring (called avocatins or avofurans), terpenoid glycosides, flavonoids, and coumarins [4,5,6,7,8,9,10,11,12]. The fruit’s flesh is pale green to bright yellow in color; is smooth, buttery in consistency, and has an exquisite flavor and aroma [13]. Although the fruit is low in carbohydrates, it is high in lipids, proteins, and minerals [12,14]. Previous consumer studies identified relevant sensory attributes that characterized avocado pulp for texture (firmness, creaminess, buttery, smoothness, and watery) and flavor (grassy, bland, nutty, and buttery) [15,16]. However, avocado cultivars are reported to differ in their chemical profiles, which can influence their sensory characteristics and consequently, their acceptability. Although consumer liking of some avocado cultivars is reported [17,18], a trained panel-guided identification of the sensory attributes that impact a particular avocado cultivar’s preference over another was not studied. The present work aimed to use predictive modeling to identify potential sensory drivers of liking for avocado pulp by evaluating the acceptability scores and sensory descriptive profiles of two commercial and five non-commercial cultivars. The study also characterized macronutrient composition, instrumental texture, and color as variables, to understand the liking and identify rapid assessment tools related to consumer acceptability.

## 2. Materials and Methods

### 2.1. Plant Material

**Figure 1:**
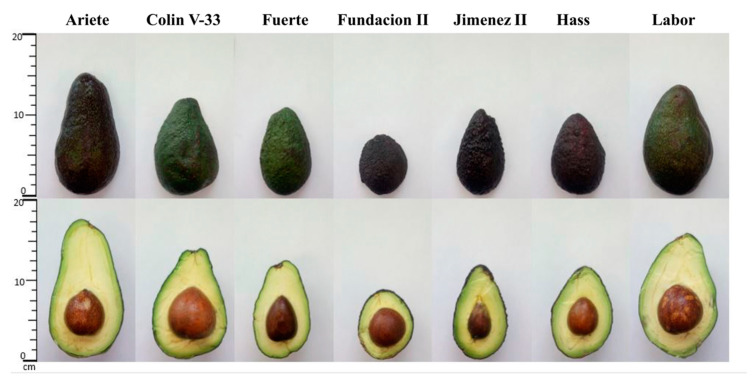
Avocado (*Persea americana*) fruits including commercial and non-commercial cultivars.

Avocado (*P. americana*) cultivars, shown in Figure 1, were hand-picked (6 October 2017) from the Fundación Salvador Sanchez Colin—(CICTAMEX) experimental field, located in Coatepec Harinas, Estado de Mexico, Mexico (18° 55′ N, 99° 45′ W, 2240 m above sea level). Collected samples (10–12 kg of each cultivar) included five cultivars from the CICTAMEX collection and specimens from two commercially marketed cultivars (Hass and Fuerte). Fruits from all evaluated cultivars were harvested from the same orchard, from different locations within a single tree, selecting those at full physiological maturity (but unripe). Samples were air shipped in closed containers with activated charcoal. Upon arrival to the Centro de Biotecnologia-FEMSA (Tecnologico de Monterrey, Monterrey, NL, Mexico), all fruits were kept at 20 °C (85–90% relative humidity) for seven days, to complete the ripening process, until reaching an optimal state for consumption. Information related to horticultural race and general phenotype description of the cultivars used in the present study is summarized in Table 1.

### 2.2. Physicochemical Analyses

The required number of avocado fruits (of each cultivar) were randomly selected to complete a sample composite of about 1.5 kg of pulp, based on their average weight and pulp content (Table 1). Avocado pulps were then manually pureed, and vacuum packaged in transparent nylon-polyethylene bags (Uline, Apodaca, NL, Mexico), containing approximately 250, 150, and 40 g of sample for their use in proximate composition, instrumental texture, and color determinations, respectively. The packaging material had a thickness of 5 µm, a standard barrier oxygen transmission rate of 63 cm^3^/m^2^ for 24 h at 23 °C, and 0% relative humidity, and a moisture vapor transmission rate of 4.8 g/m^2^, 24 h at 37 °C at 90% relative humidity. Samples were stored at 4 °C, and physicochemical analyses were conducted within the following 24 h.

#### 2.2.1. Proximate Macronutrient Composition

Moisture, protein, lipid, ash, sugar, and crude fiber content in avocado pulps were determined in triplicate, following the standard methods from the Association of Official Analytical Chemists International [23]. Total carbohydrate content was calculated by difference and dietary fiber was also determined in triplicate, using the AOAC methods 997.08 and 999.03.

#### 2.2.2. Instrumental Texture Analyses

Instrumental texture determination of avocado puree samples was conducted using a TA-XTplus (Stable Micro Systems, Godalming, UK) texture analyzer. Measurements were performed using the TTC Spreadability Rig (HDP⁄ SR) fixture, consisting of a set of male and female acrylic cones with 90° angles. Avocado puree packages were conditioned at 25 °C for 20 min before analysis. Samples were then filled into the female (lower) cone with a spatula, pressed lightly to eliminate air pockets (visible through the cone), and the surface was flattened. The female cone was fixed on the base holder of the texture analyzer. Protocol for cheese spread (Texture Exponent software version 6.1.11.0—Stable Micro Systems, Godalming, UK) was used in the determinations, with a 5 kg load cell (test speed—3.0 mm/s and post-test speed—10.0 mm/s). Distance traveled (23 mm) by the male cone was recorded, from its start point at 25 mm over the bottom of the female cone and until it was introduced into the sample, stopping when the final gap between the two cones was precisely 2 mm. Textural data were recorded as force in grams (g) versus time (s), and the software calculated the following instrumental parameters as output variables—firmness (g), work of shear (g s), stickiness (g), and work of adhesion (g s). Determinations were performed at controlled room temperature (25 °C) with five replicates per sample.

#### 2.2.3. Instrumental Color Determinations

Instrumental color of avocado pulps from each cultivar were determined with a tristimulus Minolta CR-400 colorimeter (Konica Minolta Sensing Inc., Osaka, Japan), using a D75 illuminant at an observation angle of 10°. A standard white tile was used as a calibration reference. Readings for L* (lightness), a* (red-green axis), and b* (yellow-blue axis) CIE*Lab* coordinates were recorded in five replicates (*n =* 5) for each cultivar. Variations of L*, a*, b* (∆L*, ∆a*, ∆b*), and total color difference (∆E_ab_*) were calculated for each cultivar using Hass commercial cultivar as a reference control. The following equation was used:

(1) $$∆E_{ab}^{*}=\;\sqrt{{\left(L_{1}^{*}-L_{0}^{*}\right)}^{2}+{\left(a_{1}^{*}-a_{0}^{*}\right)}^{2}+{\left(b_{1}^{*}-b_{0}^{*}\right)}^{2}}$$

where *L*_0_*, *a*_0_*, and *b*_0_* included the reference values for control (Hass cultivar) and *L*_1_*, *a*_1_*, and *b*_1_* indicated values for cultivars. Values of ∆E_ab_* > 3.5 units were considered as indicators that instrumental color differences were possibly perceived by an average observer [24]. 

### 2.3. Sensory Analyses 

#### 2.3.1. Sample Preparation

Fruits were weighed, washed, and soaked for 5 min in chlorinated water (200 ppm) for sanitization, and dried at room temperature for one hour. About 10 min before each sensory testing session (descriptive and affective tests), sample preparation was initiated; pulp was manually separated from peel and seed. Avocado fruits were randomly selected to obtain ~1 kg of pulp from each cultivar, based on their average weight and pulp content (Table 1). Pulps from each cultivar were hand-scooped and placed into plastic bags. Headspace was removed, and then the pulp was manually pureed within the bags, for 5 min, until color and texture were visually homogenous. For sensory evaluations, avocado puree samples (20 g) were placed in disposable soufflé cups (30 mL), identified with random three-digit numbers, and presented in random order to trained and untrained judges.

#### 2.3.2. Sensory Descriptive Profiling 

Ten trained panelists from SensoLab Solutions SC, a sensory and consumer science laboratory center, with over 500 h of descriptive experience in a wide variety of foods, conducted descriptive sensory profiling of nineteen sensory attributes using a 15-cm free scale. These attributes were obtained previously during two consensus sessions, where the panel as a group enlisted the most relevant attributes that characterized the studied samples. Five additional one-hour sessions were carried out to train the expert panel in the attributes that were obtained during the consensus. The ballot was designed using Fizz Forms (Biosystems, Couternon, France). All trials were conducted in individual sensory booths with white lighting and data were collected with FIZZ^®^ Acquisition software version 2.50 (Biosystemes, Couternon, France). References and samples were rated using a 15-cm universal Spectrum™ line scale with 0 cm representing “none” and 15 cm representing “strong” [25]. Samples were presented with a random three-digit code, in random, monadic sequential order, and evaluated in triplicates, on four different days. Rinsing water and crackers were provided. No information about the test or samples was given to the panelists before or during the evaluations. Attribute definitions and references used in the evaluations are shown in Table 2.

#### 2.3.3. Consumer Evaluations

Affective data were collected from *n* = 116 frequent avocado adult consumers (frequency > twice a week; 28% males; 72% females) within 18–51 years old (mean age 33.62 ± 12.73 years). Participants were previously recruited and were instructed to avoid eating or drinking anything but water at least two hours before the sensory evaluation. Consent forms provided participants with information on avocado samples. They were also asked for their willingness to participate in the study, as part of a graduate research project from the Department of Bioengineering, School of Engineering and Sciences of Tecnologico de Monterrey, Campus Monterrey, Mexico (Ethics ID: CSERDBT-0001). Participants evaluated appearance, texture, flavor, and overall liking, using a nine-level hedonic scale. The sessions were conducted at SensoLab Solutions SC, located at the Technology Transfer and Innovation Center of Tecnologico de Monterrey. Generally, 20–30 consumers participated in each evaluation session, and the study was conducted for three days. Participants received an economic incentive at the end of their participation.

### 2.4. Statistical Analysis

For physicochemical and instrumental determinations, normality of the data was evaluated by the Shapiro-Wilk test. The mean ± SE or median (interquartile range) was reported for parametric and non-parametric data, respectively. Determinations on the avocado composited samples included, proximate composition (*n* = 2), instrumental texture (*n* = 5), and instrumental color (*n* = 5), while the fruit’s weight, length, and percent pulp contents were determined from individual specimens of each cultivar (*n* = 3–5). Analysis of variance (ANOVA) and mean separations were conducted with Fisher’s least significant difference (LSD) post-hoc tests for parametric variables, and Kruskal-Wallis with Nemenyi post-hoc test for non-parametric variables. Significant differences were assessed at a *p* < 0.05. Pearson product-moment correlations for each pair of variables were also calculated to assess the relationships between sensory and physicochemical data. Statistical analyses were performed using the JMP software version 15.0 (SAS Institute, Cary, NC, USA).

For the descriptive data, ANOVA was performed using as a complete randomized design using product as a fixed effect and panelist as random effect, using Fizz Calculations software version 2.50 (Biosystems, Couternon, France). A post-hoc means comparison using Fischer’s Protected LSD at a 95% confidence level was performed to determine significant differences [26,27]. For liking analysis, one-way ANOVA was performed and Fischer’s Protected LSD as a post-hoc test at *p* < 0.05 level of significance using Fizz Calculations software version 2.50 (Biosystems, Couternon, France) [28].

External preference mapping methodology was used to relate the preferences shown by the consumers to descriptive sensory characteristics of the different avocado pulps. The first step consisted in mapping the pulps on the basis of their sensory descriptive characteristics. Principal Component Analysis (PCA) was used to construct a sensory descriptive biplot with all studied descriptive attributes (individuals run by principle means) by correlations (standardized). Components were retained if they explained at least 15% of the variance. The second step was the construction of the predictive models of external preference maps using consumer hedonic data, which were performed using the Fizz Calculations software version 2.50 (Biosystems, Couternon, France) and by the XLSTAT software (XLSTAT, 2020, Addinsoft, Germany). Consumer overall liking scores were regressed onto the product scores on the principal components of the sensory space included in the PCA biplot (obtained with the trained panel data), using a quadratic model. Quadratic surface model was selected, since it corresponded to the complete model, which allowed to take into account interactions between characteristics.

## 3. Results

### 3.1. Fruit Morphological Traits and Proximate Macronutrient Composition

Morphological traits of the sampled cultivars, as previously reported by CICTAMEX [19,20,29], were confirmed in the present work; their characteristics were documented in Table 1 and can be visualized in Figure 1. All cultivars used in the study, including Hass and Fuerte, were hybrids or selections of Mexican and Guatemalan races. The median fruit weight of non-commercial cultivar Jimenez II (195 g) was the closest in value to the weights of commercial cultivars Hass and Fuerte (198 and 237.2 g, respectively), followed by Fundacion II (190.1 g); while fruits from Colin V-33, Labor, and Ariete had average weights greater than 300 g.

Fruit length values for cultivars Colin V-33 and Jimenez II (11.8 and 10.7 cm, respectively) were not significantly different than those of commercial cultivars Hass and Fuerte (9.8 and 10.5 cm, respectively). While Ariete and Labor cultivars presented the fruits with the highest average lengths (13.6 and 15 cm, respectively). Fundacion II fruits presented the lowest length values (7.4 cm). Values of percent pulp yield are considered relevant parameters for commercial applications, since they represent the edible portion of the fruit. Pulp yields (Table 1) followed a similar trend than fruit lengths, thus the Colin V-33 and Jimenez II yield values (73.1 and 79.5%, respectively) were non-significantly different from those of commercial cultivars. While the longest cultivars Ariete and Labor had the highest median pulp yields (>76%), and Fundacion II (the shortest in length) had the lowest pulp yield (64.1%).

Proximate macronutrient composition, shown in Table 3, indicated that for five of the pulps, lipids were the primary macronutrient (>13%), closely followed by carbohydrates (>8%) and then proteins (1.3–2.2%). However, the carbohydrate contents for Jimenez II and Hass pulps were slightly higher or equal than the lipid contents, respectively. The moisture contents ranged between 54 and 73%, and the fiber and ash contents were less than 3%. Fuerte and Hass pulps contained significantly higher lipid contents (21.3 ± 0.2% and 17.7 ± 0.2%, respectively) and lower moisture levels (54.7 ± 0.2% and 57.7 ± 0.2%, respectively) than the other cultivars. Our results indicated that total lipid concentrations were inversely (*r* = −0.88, *p* = 0.009) related to moisture contents. Additionally, carbohydrate and sugar levels were both inversely and strongly correlated (*r* = −0.95, *p* < 0.0012) to moisture concentrations.

### 3.2. Descriptive Sensory Analyses of Avocado Pulps

Significant differences (LSD, *p* < 0.05) were observed for ten of the nineteen evaluated descriptors (Table 4). The sensory characteristics that differentiated the pulps the most were the attributes related to lipids’ flavor impact and texture. While Hass, Jimenez II, Fuerte, and Colin V-33 were characterized for having the highest global flavor impact, Ariete had the lowest (LSD, *p* < 0.05). Creamy flavor was perceived in the highest impact for Colin V-33, Fuerte, Hass, and Jimenez II (LSD, *p* < 0.05). In texture, Hass had the strongest creamy/oily texture perception (LSD, *p* < 0.05). Additionally, Hass, Fuerte, and Jimenez II were also characterized for having the highest texture perception in firmness, cohesiveness, and lipidic residual, while Ariete, Fundacion II, and Labor had the lowest perception (LSD, *p* < 0.05). Additionally, the fiber strands attribute was different between samples (LSD, *p* < 0.05), being significantly higher in the Fundacion II cultivar. 

**Figure 2:**
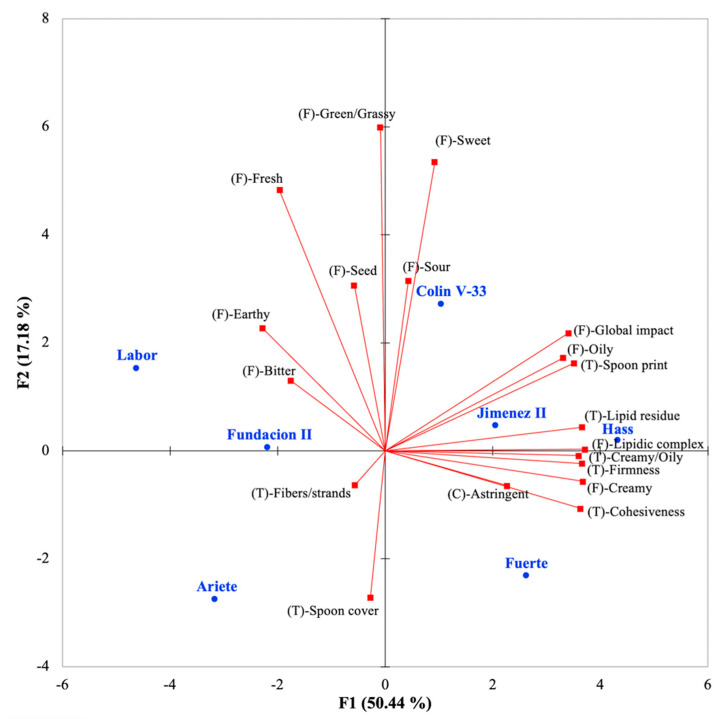
Principal component analysis (PCA) biplot of components F1 and F2, explaining 68% of the variance in the sensory descriptive profiles of seven avocado cultivars. Avocado samples are shown in blue (●), while vectors for sensory descriptive attributes are shown in red (■), and the descriptor names are shown in black. Sensory attributes were also classified with an abbreviation that indicated if they were related to flavor (F), sensory texture (T), or a sensory chemical sensation factor (C).

A sensory PCA biplot was generated with all sensory descriptive attributes (Figure 2), where the first two dimensions explained 67.6% of variability in descriptive profiles of the tested avocado pulps. Sensory attributes were related to flavor, texture, and chemical factor sensations. Sensory attributes that loaded on Component 1 included flavor attributes such as lipid complex, creamy, global impact, oily, and earthy; it also included the texture descriptors lipidic residual, firmness, cohesiveness, creamy/oily, and spoon print. Component 2 involved flavor descriptors such as green/grassy, sweet, fresh, and sour. 

### 3.3. Liking of Avocado Pulps by Consumers

The commercial preference of consumers towards the Hass cultivar was evidenced since it presented high liking scores that ranged between 7.1 and 7.2 hedonic points, as shown in Table 5. The most surprising results were obtained for the non-commercial cultivar Colin V-33 (6.9–7.2 hedonic points), since it ranked in the top liking group for all parameters and showed non-significant differences for appearance, texture, flavor, and overall liking when compared to Hass. The least overall liked cultivar was the non-commercial Fundacion II (5.6 points), and for the liking of appearance, commercial cultivar Fuerte (5.9 points) was also significantly lower (LSD, *p* < 0.05). Overall acceptability data for the rest of pulps ranged in the hedonic scale between 6.2 and 6.5 points; their values were slightly lower (but statically significant LSD, *p* < 0.05) than the most liked cultivars (Hass and Colin V-33).

### 3.4. Preference Mapping of Consumer Acceptability and Descriptive Sensory Attributes

**Figure 3:**
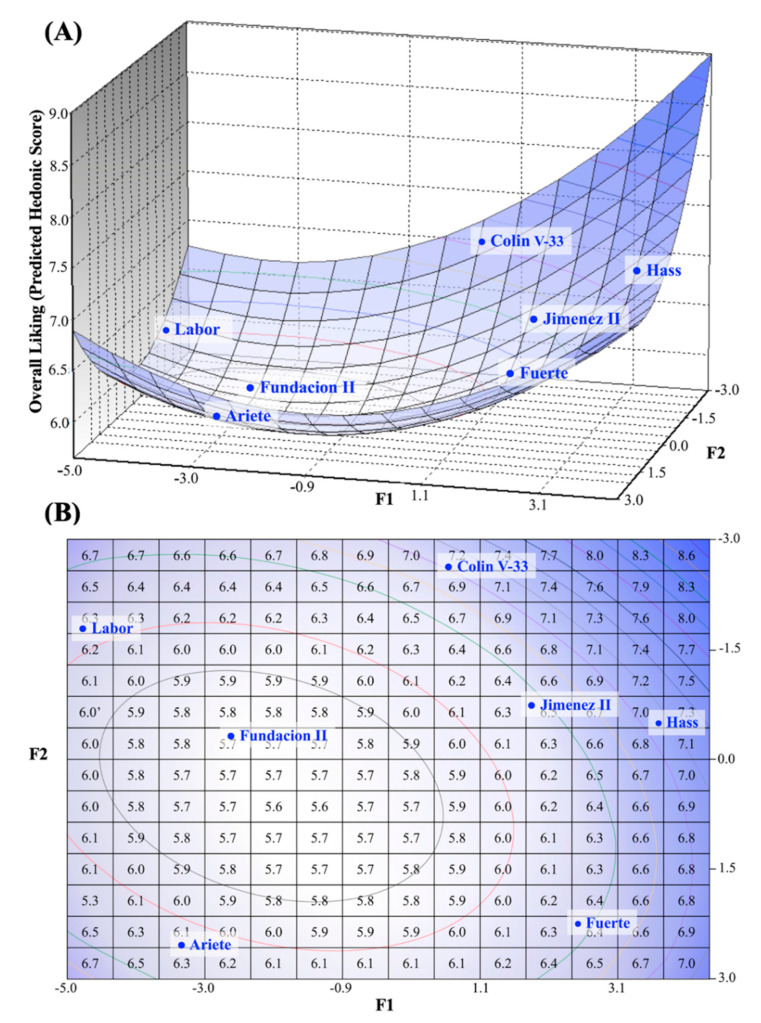
Tridimensional (**A**) and bidimensional (**B**) external preference map obtained by quadratic modeling of the overall liking of frequent avocado consumers (*n* = 116) for seven avocado cultivars, and placement of the affective data within sensory descriptive space. Principal component biplot of sensory descriptive data (components F1 and F2) used in construction of external preference map is shown in Figure 2. * Scores in the bidimensional map (B), represent the predictive hedonic values.

As previously mentioned, significant differences were observed in the liking of consumers for the seven studied cultivars. Overall acceptability scores for the seven pulps ranged from 5.6 to 7.2 in a nine-point hedonic scale, and generated three distinctive groups (LSD, *p* < 0.05). The external preference map shown in Figure 3 was obtained by modeling the overall acceptability scores over the descriptive map, where the best fit was obtained using a quadratic model (*R*^2^ = 0.98). Furthermore, the overall liking scores were highly correlated to appearance, texture, and flavor variables (*r* = 0.87, 0.96, and 0.96, respectively, *p* < 0.05). 

External preference mapping of affective data (Figure 3) was aligned with the descriptive map (Figure 2) to gain further understanding on the sensory attributes that drove the preference of the evaluated avocado pulps. According to the results, the sensory attributes that appeared responsible for the driving of like (areas shown in circles in Figure 3A,B) included the flavor descriptors of global impact, oily, and the texture attributes of creamy/oily and firmness. The alignment of descriptive and affective data in the preference map (Figure 3 and Figure S1) also provided valuable insight into which pulps were most desirable for percentages of consumers. Hass, Colin V-33, and Jimenez II samples fell in a region of the map that was characterized by high-acceptability (90–100% of satisfied consumers), followed by a region of slightly lower but still high-acceptability rates (60–80% of satisfied consumers), in which cultivar Fuerte was located. None of the avocado pulps were located in the mid-acceptability region (20–60% satisfaction) but Fundacion II, Labor, and Ariete were located in the least-liked region in which consumer satisfaction percentages ranged from 0–20%.

### 3.5. Proximate Macronutrient, Instrumental Texture, Instrumental Color, and Sensory Relationships

**Figure 4:**
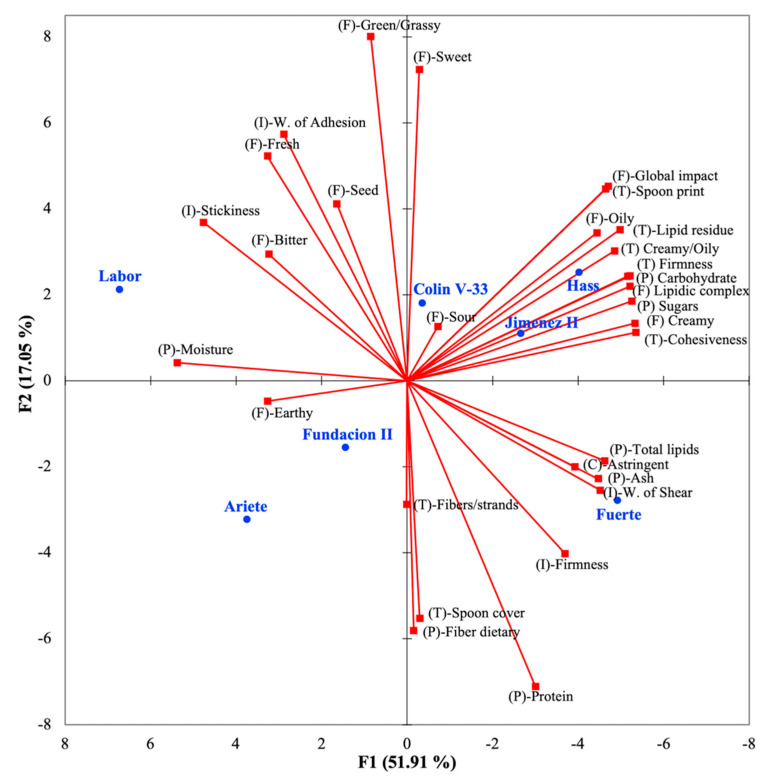
Principal component analysis (PCA) biplot of components F1 and F2, explaining 69% of the variance in the sensory descriptive profiles, proximate macronutrient composition, and instrumental texture analysis of the seven avocado cultivars. Avocado samples are shown in blue (●), while vectors for sensory descriptive attributes are shown in red (■), and the descriptor names are shown in black. Variables were also classified with an abbreviation indicating if they were related to sensory flavor (F), sensory texture (T), sensory chemical sensation factor (C), proximate macronutrient composition (P), or instrumental texture analysis (I).

The relationships between sensory affective and descriptive data, shown in Figure 3 and described in the previous section, were key to gain insight on the potential sensory drivers of liking. A second PCA was also constructed to visualize how the differences in proximate macronutrient composition and instrumental texture were related with the sensory descriptive attributes of the avocado pulps (Figure 4). As previously discussed, cultivars that were located in high-acceptability regions (Hass, Colin V-33, and Jimenez II) were associated with sensory attributes related to flavor, particularly flavors associated with lipid notes. Total carbohydrates and sugars were variables that appeared to be relevant to the differentiation of Hass, Colin V-33, and Jimenez II from other pulps, as they loaded in the same PCA quadrant (Figure 4). 

Table 3 shows the lipids to carbohydrate ratios for the pulps; this parameter indicated that when the lipid content gets higher in relation to their carbohydrate content (as for the Ariete and Labor pulps), the balance in flavor sensory attributes in the PCA quadrant seemed to move away from the desirable intensities (Figure 3). However, as shown in Figure 4, the relationship between macronutrient composition and desirable sensory descriptive profiles was not simple. Cultivar Colin V-33, which was among the most liked by consumers, had similar lipid to carbohydrate ratios than Fuerte cultivar and the least liked Fundacion II cultivar (Table 3), but Colin V-33′s sensory sweetness scores were significantly higher (Table 4).

PCA biplot shown in Figure 4 also aided in the visualization of chemical components (Table 3), sensory attributes (Table 4), and instrumental texture parameters (Table 6) that differentiated avocado pulps. Most sensory texture attributes related to lipidic sensations in the mouth, assessed by trained panelists, loaded in the quadrant with the most desirable pulps (Hass, Jimenez II, and Colin V-33). Relevant sensory texture attributes included lipidic residual, firmness, creamy/oily, and spoon print. Some instrumental texture parameters were noted to be correlated with some sensory texture descriptive attributes such as cohesiveness, which inversely correlated with instrumental stickiness (*r* = −0.75). Additionally, instrumental stickiness showed a significant (*p* = 0.01) and direct correlation with moisture contents (*r* = 0.88). As shown in Figure 4, both the stickiness and moisture vectors were characteristics associated with the least liked cultivar Labor.

The liking of commercial Fuerte cultivar was difficult to understand since it ranked in the second-best group for overall liking (Table 5 and Figure S1); however, its chemical and texture characteristics were different to those of Hass, Jimenez II, and Colin V-33. In the PCA biplot space (Figure 4), Fuerte cultivar was associated with the vectors for instrumental firmness, total lipids, and work of shear; the latter was inversely related to moisture (*r* = −0.77).

**Figure 5:**
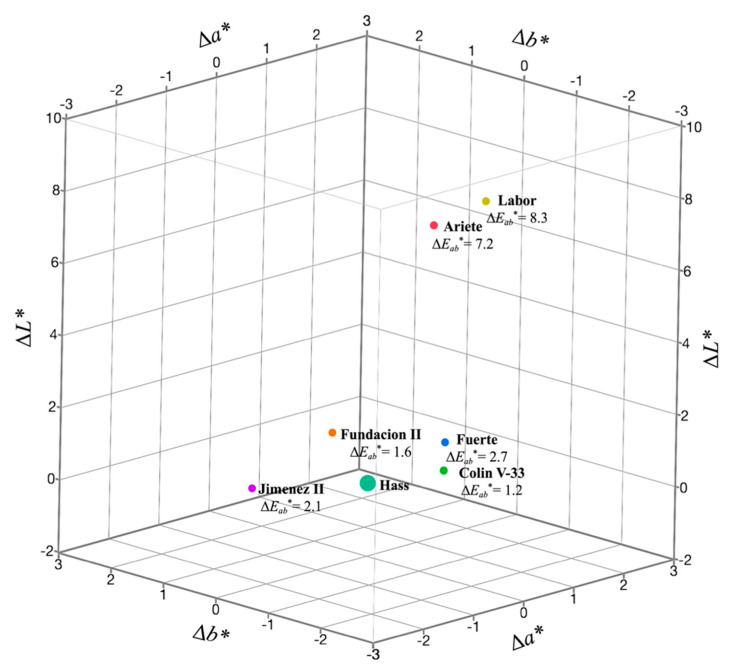
Instrumental colorimetric differences for commercial and non-commercial avocado cultivars in reference to the widely accepted Hass cultivar. Instrumental data for the Hass cultivar were used as reference control to calculate the deviations of each cultivar for the colorimetric parameters, which included ∆L*, ∆a*, ∆b*, and total color difference (∆E_ab_*).

Data on instrumental colorimetric parameters of avocado pulps were expressed as ∆L*, ∆a*, ∆b* in relation to Hass cultivar (as reference control). Instrumental color differences among avocado cultivars are shown in Figure 5 and Table S2. Color variation values (∆E_ab_*), also shown in Figure 5, were also calculated as quantitative parameters that integrated the ∆L*, ∆a*, ∆b* values. Results indicated that the least liked pulps, Labor and Ariete, presented higher ∆L* values (∆L* = +8.15 and ∆L* = +7.20, respectively) indicating higher lightness values than Hass cultivar. Colin V-33, Fuerte, Fundacion II, and Jimenez II only showed minor variations in ∆L*, denoting similarity to Hass cultivar. In contrast, ∆a* and ∆b* values, in reference to the Hass cultivar, were similar for Fuerte and the non-commercial cultivars, suggesting that the green and yellow chromaticity was similar among all pulps. Color differences (∆E_ab_*) were the instrumental parameters that differentiated samples the most from Hass and the values ranged from 1.20 to 8.31 (Figure 5). 

## 4. Discussion

### 4.1. Fruit Morphological Traits and Proximate Macronutrient Composition

Cultivars developed by the CICTAMEX foundation (Ariete, Colin V-33, Fundacion II, Jimenez II, Labor) were characterized morphologically, chemically, and compared to commercial cultivars (Hass and Fuerte) (Table 1 and Table 3). In agreement with our findings, Cajuste-Bontemps et al. [29] and Alemán-Reyes et al. [19] reported that fruits from Colín V-33 cultivar presented similar pulp yields than those form Hass and Fuerte cultivars (Table 1). However, Colin V-33 had significantly higher fruit weights; similarly, prior authors reported high fruit weights (>319 g) for the same cultivar and described it as an unfavorable commercial characteristic since the calibers of greater demand oscillate between 200 and 300 g [19,29].

Results from proximate macronutrient analyses (Table 3) indicated that all sampled cultivars (Guatemalan X Mexican hybrids) were above the California Avocado Industry standard for minimum dry matter percentages of 20.8% set for Hass [30]. According to Yahia & Woolf [31], the 20.8% dry matter standard approximates a minimum oil content of 8%. Total lipid concentrations shown in Table 3 were found to be inversely correlated (*r* = −0.88) to moisture contents. Similarly, previous research reported that during avocado fruit development, the moisture levels declined, detailed as parallel increases in dry matter and lipid contents [32]. Other researchers that focused on the chemical characterization of avocado pulps observed that high moisture and low moisture in dry fruits contained lower lipid levels, but also showed lower levels of other macronutrients such as carbohydrates, sugars, and proteins [1,30]. 

### 4.2. Descriptive Sensory Analyses of Avocado Pulps

In the present study, sensory descriptive analyses showed that the attributes that differentiated the pulps the most were related to the lipids’ impact on flavor and texture descriptors (Figure 2). In agreement, a positive correlation between oil content and palatability (flavor), as a unique sensory attribute, was previously reported for different commercial cultivars as determined by “super-critical tasters” that were very familiar with the avocado fruit and expected more of it than would the average consumer [33]. Other published sensory studies also performed the scaling of various sensory attributes in avocado samples with different chemical compositions, although not with trained panels. In a study conducted by Obenland et al. [15], avocado sensory attributes were defined by a consumer panel (*n* = 15–20), which also conducted affective testing of the same twelve avocado samples; data were used for the selection of eight main sensory attributes that were associated with the samples. All avocado samples included in their study were from the Hass cultivar, grown in different locations, and harvested on different years. The list of potential avocado descriptors was based on a previous study also conducted with the cultivar Hass [31]. Although foundational work for the selection of the eight main attributes present in avocado, with consumer evaluations, generated valuable knowledge [15]; the attributes were determined using only Hass cultivar, which might have possibly limited the sensory description of other attributes not present or pronounced in that particular cultivar. The main avocado sensory attributes identified in their work included four texture attributes (firm, creamy, buttery, smooth, and watery) and four flavor attributes (grassy, bland, nutty, and buttery). Moreover, using consumer panels, other authors confirmed the presence of similar attributes in studies with Hass avocado fruit, and in other cultivars reported as Hass hybrids [18,34]. In the aforementioned work, sensory attribute scaled were limited to a creamy texture (watery to creamy), rich (bland to rich), and grassy flavor (grassy to not grassy) on 15-cm line scales. It was not clear if the ‘richness’ definition was evaluated as an overall attribute or if it was defined for flavor or texture, but it was clearly correlated to the creamy texture attribute (*r* = 0.86) [15]. 

Literature on sensory studies conducted with avocado cultivars other than Hass and its hybrids was found to be very scarce [16,17]; and as previously mentioned, works conducted with trained descriptive panels on the evaluation of different avocado cultivars were not found. Among the few works that included various cultivars, Shaw et al. [17] conducted sensory hedonics on twenty-one avocado samples; many of them belonged to the West-Indian avocado race characterized by having lower oil contents but described as being well adapted to subtropical regions. West-Indian hybrids are therefore commercially grown in Florida, USA [35]. Consumers that evaluated the pulps documented flavor sensory descriptors, which included nutty, sweet, bitter, and mild. Formal descriptive sensory analyses with trained panels were published for the Hass cultivar samples [8,36]. However, the aims of both studies were different from the identification of drivers of liking and focused on documenting the effects of emerging technologies and storage on sensory profiles. Salgado-Cervantes et al. [36] using an experienced sensory panel identified twelve descriptors that were classified into visual appearance (homogeneity, shiny, and color), aroma (avocado, boiled vegetable, and nutty), flavor (bitter, fatty, and astringent), and texture (unctuous, grainy texture, and fibrous). The attributes were reported to be relevant sensory descriptors present in the control and flash vacuum-expansion processed avocado samples.

### 4.3. Preference Mapping of Consumer Acceptability and Descriptive Sensory Attributes

Most avocado fruits grown commercially in the world are from the Hass cultivar, since consumers are positively drawn to its taste and texture [18]. Our consumer acceptability results confirmed that Hass presented high liking scores (Table 5). Unexpectable high liking scores were also obtained for non-commercial cultivar Colin V-33, which showed non-significantly different liking scores when compared to Hass. Our results were in agreement with observations reported by López-López [37]. In their work, the authors conducted a small consumer acceptability study (*n* = 14) using three of the same cultivars evaluated herein (Hass, Fuerte, and Colin V-33). Their results indicated that hedonic scores for flavor, color, odor, and external appearance for cultivar Colin V-33 were not significantly different from those of Hass. Therefore, the observations from both independent studies confirmed that Colin V-33, a non-commercial cultivar, was highly liked by consumers.

In the present study, the external preference map was modeled using overall liking scores from consumers, followed by its placement over a previously constructed descriptive map (Figure 3). Overall liking scores were highly correlated to the appearance, texture, and flavor liking scores (*r* = 0.87, 0.96, and 0.96, respectively, *p* < 0.05). It is possible that consumers being untrained assessors were not able to accurately differentiate appearance, flavor, and texture but they clearly indicated that the three attributes were relevant for consumer satisfaction of avocado fruit. Pereira et al. [16] also observed for avocado samples that it is common in consumer research to observe correlations among scores for overall liking with those for the liking of specific attributes; possibly because of the halo effect, since consumers are more focused on the general affective response and when they like or dislike a sample, they tend to give similar scores for all its attributes. Obenland et al. [15] conducted a follow-up of the changes in sensory attributes and hedonics during maturation of Hass cultivar from different locations and harvesting years. The aforementioned study showed that liking declined when the texture descriptor for creaminess declined and the flavor descriptor for grassiness increased, indicating that both flavor and texture attributes contributed to liking. Our results confirmed and complemented these observations, since flavor and texture sensory attributes were found to be the drivers of liking; but more specifically flavor descriptors such as lipidic notes, sweetness, and some fresh/green notes, together with texture descriptors for firmness, creaminess, and lipidic residue. The present work also characterized bitter and astringent as sensory attributes present in the avocado pulps, in agreement with descriptive work on avocados conducted by Salgado-Cervantes et al. [36]. Statistical comparisons among pulps did not show marked differences for bitterness or astringency (Table 4), but in the preference map (Figure 2 and Figure 3) both attributes were associated with pulps that were penalized in liking (Labor and Fuerte). Sensory flavor is complex, and for the present work it was limited to the studied attributes, therefore it is possible that those particular pulps transmitted sensations that require further descriptive work.

### 4.4. Proximate Macronutrient, Instrumental Texture, Instrumental Color, and Sensory Relationships

As previously discussed, total lipids are widely reported in the literature to be a desirable quality in avocado fruit [30]. In this work, all studied cultivars were Guatemalan X Mexican hybrids, and their proximate composition indicated that all were above the California Avocado Industry standard for minimum dry matter percentage (20.8% set for Hass) [30]. However, in the present work we were able to observe that a balance between carbohydrates, sugars, and lipids appears to be relevant to avocado sensory flavor profile (Figure 4). An observation that was also supported by direct slight correlations between affective flavor liking with carbohydrate and sugar contents of the pulps (*r* = 0.65 and 0.61, respectively).

In their work with Hass cultivar, Obenland et al. [15] concluded that carbohydrates, because of their low concentrations, might not influence acceptability. However, their observations could be limited by the use of that single cultivar. A prior study conducted with various cultivars, including different avocado races, focused on carbohydrates [17], and showed that the West-Indian cultivars contained higher levels of seven carbon (C7) sugars, which are rare in nature but are present in avocado fruit. The C7 sugars D-manno-heptulose and perseitol were the main sugars present in some cultivars of the West-Indian race background. Furthermore, West-Indian race fruits contained higher concentrations of the C7 sugars than those for glucose and fructose, and the consumer panel associated them with the sweet sensory attribute [17]. In the present work, the concentrations of individual C7 sugars were not measured; therefore, we were not able to confirm prior author conclusions that when the Mexican race was present in the genetic background, the C7 sugar levels tended to be low [17]. In this study, results from total sugars concentrations were significantly higher for some of the pulps with higher liking scores (Hass and Jimenez II), however, Colin V-33 had lower sugar levels but a higher sweetness sensory scores. Perhaps further work on the characterization of individual sugar profiles, including C7 sugars, can provide further insight into the sensory observations. Flavor metabolites were also reported to play relevant roles in the generation of desirables profiles, and they can be generated from both lipids and carbohydrates, particularly sugars. Lipid degradation products such as acetaldehyde, methyl acetate, 2,4 heptadienal were associated to have high preference values [15]; nonetheless, avocado sugar metabolites are least known. Prior studies described the disappearance of C7 sugars during ripening [1,38] and suggested a potential role in the generation of flavor metabolites.

In addition to flavor, sensory texture and appearance attributes need to be considered among the potential drivers of liking of avocado pulp. Thus, in this study, sensory texture attributes clearly differentiated avocado pulps and were related to the descriptors of lipidic oral sensations. Similarly, other studies confirmed the relationship between texture attributes such as firm, creamy, smooth, and high hedonic scores [15,34]. Herein, instrumental texture measurements alone were poorly correlated to consumer liking, possibly because liking is a complex variable and is difficult to relate to individual instrumental parameters. Nevertheless, instrumental data were useful as an additional objective assessment of the characteristics of the avocado pulps evaluated. Sensory firmness was directly related to instrumental weight of shear (*r* = 0.64), and inversely to instrumental stickiness (*r* = −0.69). Similarly, sensory cohesiveness (rated higher in the most liked pulps) was also inversely related to instrumental stickiness (*r* = −0.76). These correlations served to reassure that the train panel assessments were in accordance with the texture lexicon definitions, since other authors reported similar sensory and instrumental texture relationships for semi-solid matrixes [39].

Interestingly, correlations between sensory texture attributes evaluated by a trained panel, and proximate compositions were even stronger than those for instrumental texture measurements. For instance, sensory cohesiveness showed a significant (*p* = 0.0002) and strong inverse correlation with moisture contents (*r* = −0.97). Additionally, sensory cohesiveness was strongly correlated with total carbohydrates (*r* = 0.96) and sugars (*r* = 0.95), and mildly correlated with lipids (*r* = 0.86), confirming the relevant relationship of both macronutrients to texture, in addition to flavor. Data from both sensory assessments (liking and descriptive) indicated that high moisture levels, thus lower dry matter, lipids, carbohydrates, and sugars moved the texture away from the desired sensations. Contrary to sensory cohesiveness, instrumental stickiness loaded in the same PCA quadrant of the less desirable traits (Figure 4) and was also found to be directly correlated to moisture (*r* = 0.88) and inversely to lipid content (*r* = −0.79). Therefore, results indicated that stickiness was considered as an interesting instrumental texture parameter, since it was also described by prior authors as an undesirable trait for semi-solid matrixes, such as fat spreads [40]. 

Considering lineage information shown in Table 1, it was observed that non-commercial cultivars that were located in the high-acceptability regions (Colin V-33 and Jimenez II) had common lineages with at least one commercial cultivar (Hass or Fuerte); therefore, suggesting that the progenitors were already selected for the desirable traits, such as flavor and texture. Selection was possibly performed considering high dry matter and oil contents since both chemical traits are known to drive acceptability [33]. Energy concentrations (kcal/100 g fresh weight (FW)), which served as a combined measurement of the contribution of lipids, carbohydrates, and proteins to the overall composition of the pulps are also included in Table 3, and the results clearly indicated that the most liked cultivars contained the highest values (201.3–267.2 kcal/100 g FW). However, results obtained for the Fuerte cultivar indicated that other factors could also influence liking. The Fuerte cultivar ranked in the second-best group for overall liking (Table 5 and Figure S1); although its dry matter, lipid, and caloric contents were the highest of all (Table 3). Sensory texture characteristics of Fuerte were not very different from those of Hass, Jimenez II, and Colin V-33, although the instrumental texture parameters indicated significantly higher values for its work of shear, work of adhesion, and stickiness (Table 6). It is also possible that its acceptability was slightly penalized because of its visual aspects, since its liking of appearance score by consumers were significantly lower (5.9 in a nine-levels hedonic scale, Table 5). Using Hass as a reference, the ∆a* and ∆b* values were similar for Fuerte and the non-commercial cultivars, but the ∆E_ab_* values showed some differences among the pulps (Figure 5). Labor (∆E_ab_* = 8.31) and Ariete (∆E_ab_* = 7.25) pulps showed the highest color differences, while Fuerte (∆E_ab_* = 2.7) color variation was not as high. However, Fuerte was slightly different from Hass compared to the more liked Colin-V33 (∆E_ab_* = 1.2) and Jimenez II (∆E_ab_* = 2.1). Perhaps that slight color difference was sufficient to penalize the liking of Fuerte for appearance. However, Ghidouche et al. [24] observed that ∆E_ab_*values greater than 3.5 units were required to perceive a color difference by an average observer. Labor and Ariete pulps presented ∆E_ab_*values greater than 3.5 from Hass (8.3 and 7.25, respectively), which might have partly influenced consumers’ slight overall dislike.

## 5. Conclusions

For the first time, the development of highly detailed descriptive profiles of different commercial and non-commercial avocado cultivars generated new knowledge on key sensory attributes that drove the liking for avocado pulp conveyed by consumers. Our results confirmed observations obtained from prior consumer evaluations, in which flavor and texture sensory attributes were concluded to be key for liking. Furthermore, the present study’s sensory-driven strategy generated an external preference map that facilitated the identification of sensory descriptors, which influenced the overall liking. In general, consumers tend to prefer avocados with a strong global impact, a creamy and oily flavor attributes, and other relevant sensory texture attributes that grouped in Hass’s region (one of the most preferred pulps). A non-commercial cultivar, Colin V-33, presented sweet and green notes that also appear to drive preference. Therefore, the results indicated that the drivers of liking for avocado pulp include specific lipid flavor notes, sweetness, green notes, and textures of creaminess/oiliness, lipid residue, firmness, and cohesiveness. The earthy, bitter notes, absence of fibers, and a balanced green color also complemented specific cultivars’ preferences. The role of avocado sugars in flavor remains to be further explored since the fruit contains unique carbohydrates. The present work also generated new knowledge and ideas on the possible drivers of disliking, such as stickiness, differences in color, and possibly other unexplored flavors and chemical sensations that remain to be characterized. However, results from the preference map generated valuable information that can be used by avocado breeders and processors as sensory-guided insight to develop and select cultivars with high acceptability for their commercialization strategies.

## Figures and Tables

**Table 1:** Morphological traits and genotype relationships of avocado cultivar samples at commercial ripeness.

Cultivar	Race ^1,2^	Parentage/ Origin ^2^	Fruit Main Phenotype Description	Fruit Weight (g) ^3^	Pulp Yield (%) ^3,4^	Fruit Length (cm) ^3^
Ariete	M X G	Colin V-33/ Mexico	Ripe fruit is dark green, cream-colored pulp, and elliptical shaped seed [19].	441.8 (402.2–563.7) *^a^*	76.1 (75.8–76.1) *^a^*	15.0 ± 0.3 *^a^*
Colin V-33	M X G	Fuerte/ Mexico	Ripe fruit is dark green, cream-colored pulp, and triangular-shaped seed [19].	319.4 (274.1–400.9) *^a^*	73.9 (61.4–74.6) *^b^*	11.8 ± 0.5 *^c^*
Fuerte	M X G	Unknown/ Mexico	Ripe fruit is green, thick hull, cream-colored pulp, and triangular-shaped seed [20].	237.2 (201.4–274.4) *^b^*	73.6 (63.6–73.6) *^b^*	10.5 ± 0.4 *^bc^*
Fundacion II	M X G	Hass/ Mexico	Ripe fruit is dark purple color, cream-colored pulp, and circular shape seed [19].	190.1 (172.5–199.65) *^c^*	64.1 (57.1–64.1) *^c^*	7.4 ± 0.2 *^d^*
Hass	M X G	Unknown/ USA	Ripe fruit is dark purple, cream-colored pulp, with a smooth and creamy texture, and seed of small to medium circular shape [20].	198.0 (161.1–234.4) *^b^*	69.6 (68.2–69.9) *^b^*	9.8 ± 0.4 *^c^*
Jimenez II	M X G	Hass mutant/ Mexico	The fruit has a rough, leathery hull and not adhered to pulp, black color in the ripe stage [19].	195.0 (168.1–236.3) *^b^*	79.5 (60.1–79.5) *^b^*	10.7 ± 0.3 *^bc^*
Labor	M X G	Hass/ Mexico	Ripe fruit is dark green, cream-colored pulp with circular shaped seed [20].	336.0 (323.6–497.45) *^a^*	77.6 (69.4–77.7) *^a^*	13.6 ± 0.2 *^ab^*

^1^ Race: M, (Mexican, *Persea americana var. drymifolia*), G (Guatemalan, *P. americana var. guatemalensis*).^2^ Genotype assignation, parentage, and origin reported by López-López, Barrientos-Priego, & Ben Ya’acov [21]; Rodríguez-López, Hernández-Brenes, & Díaz De La Garza [22]; Rendón-Anaya et al. [2]. ^3^ Values represent median (interquartile range) or mean ± SE for non-parametric or parametric data, respectively (*n* = 3–5). Different letters within the same column indicate significant differences, according to Kruskal-Wallis or LSD post-hoc test, for non-parametric or parametric data, respectively (*p* < 0.05); ^4^ g of pulp/100 g of total fruit’s weight.

**Table 2:** Sensory attributes, definitions, and references used in the descriptive analyses of avocado pulp.

Attribute	Definition	Reference ^1^ (Brand)
AROMATIC FLAVORS		
Global impact	Maximum flavor intensity reached by the product.	Soybean oil (Nutrioli)
Lipidic complex	Flavor associated with any kind of fats.	Soybean oil (Nutrioli)
Creamy	Naturally occurring oil that binds flavors without tasting oily by a cream perception.	Mexican creole avocado puree with 5% heavy cream (Lala).
Oily	Flavor associated with oil.	Mayonnaise (Hellmann’s)
Green/grassy	Aromatic characteristic of freshly cut leaves, grass, or green vegetables.	Freshly cut grass and Fresh lettuce
Fresh	Flavor associated with freshness.	Fresh lettuce
Seed	Character associated with chewing on seeds.	Avocado seed grinded
Earthy	A lingering earthy, musty flavor.	Sliced fresh mushroom
BASIC TASTES		
Sweet	A fundamental taste of sucrose in water is typical.	2% sucrose solution
Sour	A fundamental taste of citric acid in water is typical.	0.05% citric acid solution
Bitter	A fundamental taste of caffeine in water is typical.	0.01% caffeine solution
CHEMICAL FEELING FACTOR			
Astringent	Complex of drying, puckering, and shrinking sensations in the lower oral cavity.	Grape Juice (Welch’s)
TEXTURE			
Creamy/oily	Creamy or oily sensation in mouth.	Mayonnaise (Hellmann’s)
Cohesiveness	Degree to which sample holds together in a mass.	Banana baby puree (Gerber)
Firmness	Degree of resistance to flow	Miracle Whip (Kraft-foods)
Fibers/strands	The degree to which fibers are present.	Mexican creole avocado puree
Spoon cover	Quantity of sample attached to the outer spoon surface when compressed against sample.	Heavy cream (Lala)
Spoon print	Print left by compressing a spoon in the sample.	Table cream (Nestle)
Lipid residue	Residual oily sensation after product is swallowed (oily residual).	Mayonnaise (Hellmann’s)

^1^ References were prepared approximately 24 h before a testing session, refrigerated overnight, and removed from the refrigerator 1 h before a testing session. Intensity based on a 15-point numerical scale, where 0 represents absence and 15 represents extremely strong. Avocado creole (unknown landrace) common in Northern Mexico was obtained from a local supermarket (Monterrey, NL, Mexico) and was used for calibration purposes, and reference intensities were established by the panel consensus.

**Table 3:** Proximate macronutrient concentrations (g/100 g fresh weight (FW)) of seven avocado cultivars, including commercial and non-commercial samples.

Parameter	Ariete		Colin V-33		Fuerte		Fundacion II		Hass		Jimenez II		Labor	
Moisture	* 69.6	±0.0 *^b^*	65.9	±0.1 *^c^*	54.7	±0.2 *^e^*	66.6	±0.6 *^c^*	57.7	±0.2 *^d^*	58.4	±0.1 *^d^*	73.0	±0.0 *^a^*
Proteins	1.9	±0.0 *^b^*	1.6	±0.0 *^c^*	2.2	±0.1 *^a^*	1.9	±0.1 *^b^*	1.8	±0.0 *^b^*	1.5	±0.0 *^c^*	1.3	±0.0 *^d^*
Lipids	15.1	±0.1 *^e^*	16.0	±0.0 *^d^*	21.3	±0.2 *^a^*	13.8	±0.2 *^f^*	17.7	±0.1 *^b^*	17.0	±0.1 *^c^*	13.9	±0.0 *^f^*
Carbohydrates (CHOs)	8.4	±0.1 *^c^*	12.8	±0.1 *^b^*	16.6	±0.2 *^a^*	11.2	±1.8 *^b^*	17.8	±0.4 *^a^*	18.1	±0.1 *^a^*	8.6	±0.0 *^c^*
Sugars	2.8	±0.2 *^d^*	3.9	±0.0 *^c^*	5.0	±0.0 *^b^*	3.9	±0.0 *^c^*	5.3	±0.1 *^a^*	5.2	±0.1 *^ab^*	2.7	±0.1 *^d^*
Fiber dietary	2.9	±0.0 *^a^*	2.1	±0.0 *^c^*	2.1	±0.0 *^bc^*	1.9	±0.1 *^d^*	2.0	±0.0 *^cd^*	2.2	±0.0 *^b^*	1.7	±0.0 *^e^*
Ash	2.1	±0.0 *^c^*	1.7	±0.0 *^d^*	3.0	±0.1 *^a^*	2.7	±0.0 *^b^*	3.0	±0.0 *^a^*	2.8	±0.1 *^b^*	1.5	±0.0 *^e^*
Ratio Lipids/CHOs	1.8	±0.0 *^a^*	1.3	±0.0 *^bc^*	1.3	±0.1 *^b^*	1.3	±0.2 *^bc^*	1.0	±0.0 *^cd^*	0.9	±0.1 *^d^*	1.6	±0.0 *^a^*
Energy (kcal/100 g FW)	176.7	±0.1 *^e^*	201.3	±0.4 *^d^*	267.2	±0.3 *^a^*	176.5	±4.8 *^e^*	237.9	±0.5 *^b^*	231.2	±1.0 *^c^*	165.0	±0.2 *^f^*

* Values represent mean ± SE (*n* = 2). Different letters within the same row indicate that the means are significantly different, according to the LSD test (*p* < 0.05).

**Table 4:** Intensity ratings of nineteen descriptive attributes for seven commercial and non-commercial avocado cultivars.

Descriptor	Ariete		Colin V-33		Fuerte		Fundacion II		Hass		Jimenez II		Labor	
(F) ^1^ Global impact	* 2.3	±0.1 *^d^*	2.7	±0.1 *^ab^*	2.6	±0.1 *^ab^*	2.5	±0.1 *^bc^*	2.8	±0.1 *^a^*	2.7	±0.1 *^ab^*	2.4	±0.1 *^cd^*
(F) Lipidic complex	2.1	±0.1 *^cd^*	2.4	±0.1 *^b^*	2.5	±0.1 *^ab^*	2.2	±0.1 *^c^*	2.7	±0.1 *^a^*	2.5	±0.1 *^ab^*	1.9	±0.1 *^d^*
(F) Creamy	1.8	±0.1 *^b^*	2.2	±0.1 *^a^*	2.4	±0.1 *^a^*	1.8	±0.1 *^b^*	2.4	±0.1 *^a^*	2.4	±0.1 *^a^*	1.6	±0.1 *^b^*
(F) Oily	1.8	±0.1 *^cd^*	2.3	±0.1 *^ab^*	2.0	±0.1 *^bc^*	2.0	±0.1 *^bc^*	2.3	±0.1 *^a^*	2.1	±0.1 *^ab^*	1.6	±0.1 *^d^*
(F) Green/Grassy	2.0	±0.1 *^c^*	2.3	±0.1 *^ab^*	2.1	±0.1 *^bc^*	2.2	±0.1 *^abc^*	2.2	±0.1 *^abc^*	2.3	±0.1 *^ab^*	2.3	±0.1 *^a^*
(F) Fresh	1.4	±0.1 *^b^*	1.6	±0.1 *^ab^*	1.4	±0.1 *^b^*	1.6	±0.1 *^ab^*	1.5	±0.1 *^b^*	1.6	±0.1 *^ab^*	1.8	±0.1 *^a^*
(F) Seed	1.6	±0.1 *^ab^*	1.7	±0.1 *^a^*	1.3	±0.1 *^b^*	1.5	±0.1 *^ab^*	1.5	±0.1 *^ab^*	1.7	±0.1 *^a^*	1.6	±0.1 *^ab^*
(F) Earthy	1.0	±0.1 *^a^*	1.0	±0.1 *^a^*	0.8	±0.1 *^a^*	1.0	±0.1 *^a^*	0.8	±0.1 *^a^*	0.9	±0.1 *^a^*	0.9	±0.1 *^a^*
(F) Sweet	0.6	±0.1 *^b^*	1.0	±0.1 *^a^*	0.7	±0.1 *^b^*	0.7	±0.1 *^b^*	0.8	±0.1 *^ab^*	0.7	±0.1 *^b^*	0.8	±0.2 *^ab^*
(F) Sour	0.1	±0.0 *^b^*	0.2	±0.0 *^a^*	0.1	±0.0 *^ab^*	0.1	±0.0 *^ab^*	0.1	±0.0 *^ab^*	0.1	±0.0 *^ab^*	0.1	±0.0 *^ab^*
(F) Bitter	0.2	±0.0 *^a^*	0.2	±0.0 *^a^*	0.2	±0.0 *^a^*	0.2	±0.0 *^a^*	0.2	±0.1 *^a^*	0.2	±0.0 *^a^*	0.2	±0.0 *^a^*
(C) Astringent	1.9	±0.1 *^ab^*	2.0	±0.1 *^ab^*	2.2	±0.1 *^a^*	1.9	±0.1 *^ab^*	1.9	±0.2 *^a^*	2.0	±0.1 *^ab^*	1.8	±0.1 *^b^*
(T) Creamy/Oily	3.2	±0.2 *^cd^*	3.7	±0.2 *^bc^*	3.9	±0.3 *^b^*	3.2	±0.2 *^cd^*	4.6	±0.2 *^a^*	3.8	±0.3 *^b^*	2.9	±0.2 *^d^*
(T) Cohesiveness	4.8	±0.3 *^c^*	5.6	±0.3 *^b^*	6.8	±0.3 *^a^*	5.0	±0.2 *^bc^*	7.0	±0.2 *^a^*	6.4	±0.2 *^a^*	4.4	±0.2 *^c^*
(T) Firmness	4.7	±0.3 *^cd^*	5.4	±0.3 *^bc^*	6.0	±0.3 *^ab^*	5.0	±0.2 *^cd^*	6.5	±0.3 *^a^*	6.1	±0.3 *^ab^*	4.6	±0.2 *^d^*
(T) Fibers/strands	3.5	±0.4 *^bc^*	3.3	±0.4 *^c^*	4.2	±0.4 *^b^*	6.7	±0.3 *^a^*	4.2	±0.4 *^b^*	3.4	±0.4 *^c^*	3.7	±0.4 *^bc^*
(T) Spoon cover	6.6	±0.5 *^a^*	6.2	±0.5 *^ab^*	6.6	±0.5 *^a^*	5.6	±0.4 *^ab^*	5.1	±0.5 *^b^*	6.4	±0.5 *^a^*	5.8	±0.4 *^ab^*
(T) Spoon print	10.3	±0.5 *^c^*	11.4	±0.5 *^abc^*	11.0	±0.7 *^abc^*	10.5	±0.5 *^bc^*	11.8	±0.6 *^a^*	11.7	±0.4 *^ab^*	10.1	±0.5 *^c^*
(T) Lipid residue	3.1	±0.3 *^c^*	3.9	±0.3 *^b^*	4.1	±0.3 *^ab^*	3.1	±0.2 *^c^*	4.6	±0.2 *^a^*	4.0	±0.2 *^ab^*	3.1	±0.2 *^c^*

^1^ Letters in parenthesis indicate attribute type designated as flavor (F), texture (T), and chemical factor sensation (C). * Values represent mean ± SE (10 trained panelists by triplicate, *n* = 30). Different letters within the same row indicate that the means are significantly different, according to LSD test (*p* < 0.05).

**Table 5:** Consumer acceptability scores for overall liking, flavor, appearance, and texture of seven avocado cultivars; including commercial and non-commercial samples.

Descriptor	Ariete		Colin V-33		Fuerte		Fundacion II		Hass		Jimenez II		Labor	
Appearance liking	* 6.2	±0.2 *^cd^*	7.2	±0.1 *^a^*	5.9	±0.2 *^d^*	5.8	±0.2 *^d^*	7.1	±0.1 *^ab^*	6.6	±0.2 *^c^*	6.6	±0.2 *^bc^*
Texture liking	6.4	±0.2 *^b^*	7.2	±0.1 *^a^*	6.4	±0.2 *^b^*	5.5	±0.2 *^c^*	7.1	±0.1 *^a^*	6.3	±0.2 *^b^*	6.5	±0.1 *^b^*
Flavor liking	6.2	±0.2 *^cd^*	6.9	±0.2 *^ab^*	6.4	±0.2 *^cd^*	5.8	±0.2 *^e^*	7.3	±0.1 *^a^*	6.5	±0.2 *^bc^*	6.0	±0.2 *^de^*
Overall liking	6.3	±0.2 *^b^*	7.1	±0.1 *^a^*	6.4	±0.2 *^b^*	5.6	±0.2 *^c^*	7.2	±0.1 *^a^*	6.5	±0.2 *^b^*	6.2	±0.2 *^b^*

* Values represent mean ± SE (*n* = 116). Different letters within the same row indicate that means are significantly different, according to the LSD test (*p* < 0.05).

**Table 6:** Instrumental texture parameters of seven commercial and no commercial avocado cultivars.

Cultivar	Firmness (g)	Work of Shear (g s)		Stickiness (g)		Work of Adhesion (g s)	
Ariete	* 433.6 (411.7–444.1) *^b^*	* 414.4	±6.4 *^d^*	−418.1	±3.2 *^b^*	−125.6	±2.7 *^c^*
Colin V-33	543.7 (499.6–590.4) *^a^*	546.1	±22.7 *^b^*	−480.5	±11.8 *^c^*	−147.3	±4.1 *^c^*
Fuerte	594.9 (570.3–618.6) *^a^*	626.9	±18.1 *^a^*	−653.6	±6.3 *^f^*	−170.5	±4.1 *^d^*
Fundacion II	568.8 (503.3–604.6) *^a^*	560.0	±24.2 *^b^*	−512.9	±5.6 *^d^*	−155.2	±6.2 *^c^*
Hass	434.5 (418.0–442.3) *^b^*	486.2	±12.4 *^c^*	−480.5	±7.6 *^c^*	−112.9	±2.7 *^b^*
Jimenez II	507.8 (498.5–532.6) *^a^*	574.2	±16.1 *^b^*	−55.3	±7.9 *^e^*	−127.3	±6.9 *^b^*
Labor	315.2 (307.2–339.1) *^c^*	331.6	±11.1 *^e^*	−323.7	±6.5 *^a^*	−94.3	±4.9 *^a^*

* Values represent median (interquartile range) and mean ± SE for nonparametric and parametric data, respectively (*n* = 5). Different letters within the same column indicate significant difference, according to the Kruskal-Wallis or LSD post-hoc test, respectively (*p* < 0.05).

## Data Availability

Data is contained within the article or supplementary material.

## Supplementary Materials

The following are available online at https://www.mdpi.com/2304-8158/10/1/99/s1, Figure S1. Bidimensional external preference map obtained by quadratic modeling of the overall liking of frequent avocado consumers (*n* = 116) for seven avocado cultivars, and placement of affective data within the sensory descriptive space. Table S1. Instrumental colorimetric values of seven commercial and non-commercial avocado cultivar. Table S2. Differences in instrumental colorimetric parameters of commercial and non-commercial avocado cultivars in reference to the values obtained for cultivar Hass.
