# Supplementary material for: Insights into Drivers of Liking for Avocado Pulp (Persea americana): Integration of Descriptive Variables and Predictive Modeling
Source: Foods. 2021 Jan 6;10(1):99. doi: 10.3390/foods10010099 (PMC7825017; doi:10.3390/foods10010099)
Supplement: Supplementary file 1 [file foods-10-00099-s001.pdf]

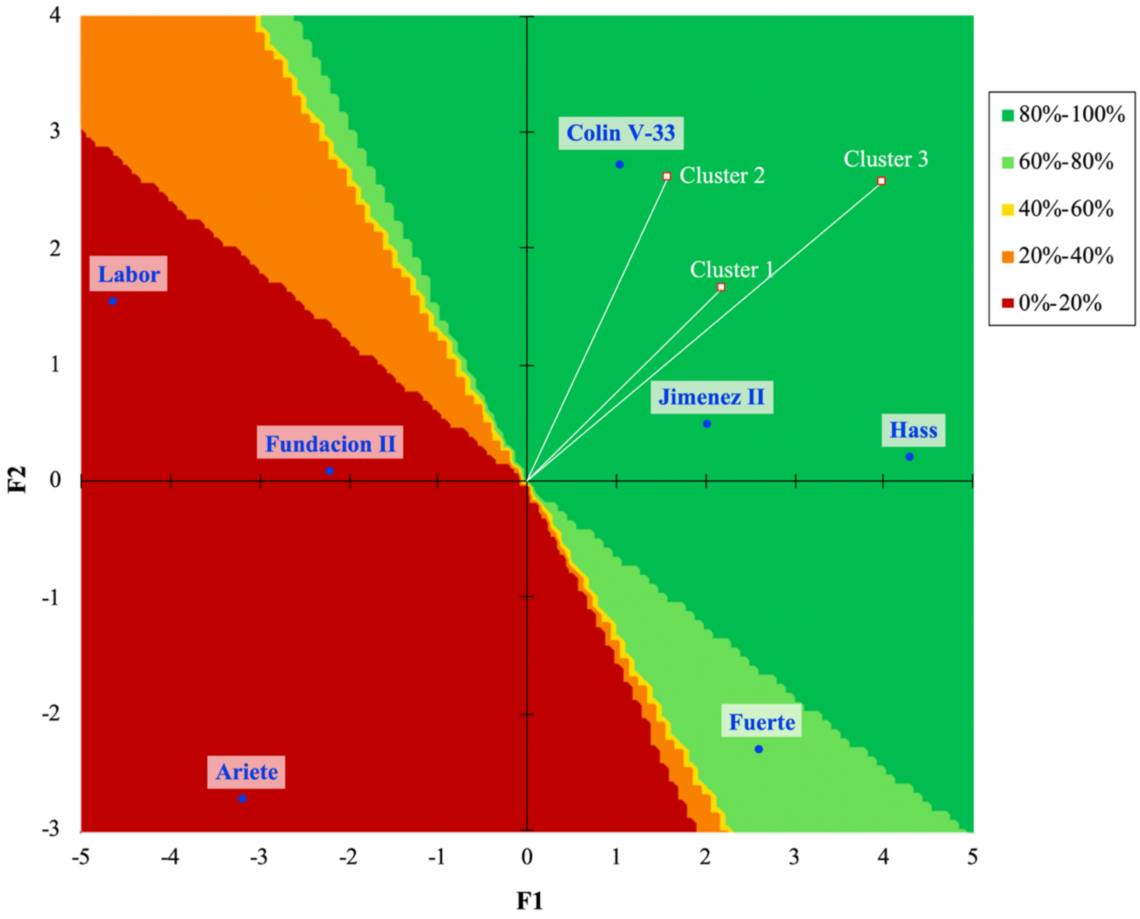

Figure S1. Bidimensional external preference map obtained by quadratic modeling the overall liking of frequent avocado consumers (*n*= 116) for seven avocado cultivars, and placement of affective data within the sensory descriptive space. Principal component biplot of sensory descriptive data used in construction of external preference map is shown in Figure 2. Color coding of regions in the map indicate the percent of consumers in each liking category (green regions indicate higher satisfaction, and red least satisfaction).

Table S1. Instrumental colorimetric values of seven commercial and non-commercial avocado cultivar

Parameter	Ariete	Colin V-33	Fuerte	Fundacion II	Hass	Jimenez II	Labor
L*	*68.1 ± 0.1 [b]	60.9 ± 0.5 [cd]	60.6 ± 0.2 [de]	61.4 ± 0.2 [c]	60.9 ± 0.3 [cd]	60.0 ± 0.2 [e]	69.0 ± 0.1 [a]
a*	-7.6 ± 0.0 [c]	-7.0 ± 0.2 [b]	-5.6 ± 0.1 [a]	-7.4 ± 0.2 [c]	-8.1 ± 0.1 [d]	-8.4 ± 0.1 [d]	-7.4 ± 0.2 [c]
b*	35.7 ± 0.1 [e]	35.9 ± 0.3 [de]	37.2 ± 0.1 [c]	37.7 ± 0.2 [b]	36.3 ± 0.1 [d]	38.2 ± 0.1 [a]	34.8 ± 0.2 [f]
Chroma	36.5 ± 0.1 [d]	36.6 ± 0.3 [d]	37.6 ± 0.1 [c]	38.4 ± 0.2 [b]	37.2 ± 0.1 [c]	39.1 ± 0.1 [a]	35.6 ± 0.1 [e]
Hue	102.0 ± 0.0 [b]	101.0 ± 0.3 [c]	98.6 ± 0.2 [d]	101.2 ± 0.3 [c]	102.6 ± 0.1 [a]	102.4 ± 0.1 [ab]	102.0 ± 0.3 [b]

*Values represent mean ± SE (n= 5). Different letters within the same row indicate that means are significantly different, according to LSD test ($p < 0.05$).

Table S2. Differences in instrumental colorimetric parameters of commercial and non-commercial avocado cultivars in reference to values obtained for cultivar Hass.

Parameter	Ariete	Colin V-33	Fuerte	Fundacion II	Jimenez II	Labor
ΔL^*	+7.20	+0.06	-0.22	+0.56	-0.83	+8.15
Δa^*	+0.56	+1.14	+2.51	+0.71	-0.29	+0.72
Δb^*	-0.64	-0.36	+0.92	+1.36	+1.92	-1.47
ΔE_{ab}^*	7.25	1.20	2.68	1.63	2.11	8.31
